# Beneficial effect of heat-killed *Lactiplantibacillus plantarum* L-137 on intestinal barrier function of rat small intestinal epithelial cells

**DOI:** 10.1038/s41598-024-62657-0

**Published:** 2024-05-29

**Authors:** Mototsugu Watanabe, Hiroko Nakai, Tatsuya Ohara, Kengo Kawasaki, Shinji Murosaki, Yoshitaka Hirose

**Affiliations:** Research & Development Institute, House Wellness Foods Corporation, 3-20 Imoji, Itami, Hyogo 664-0011 Japan

**Keywords:** *Lactiplantibacillus plantarum* L-137, Intestinal permeability, Tight junctions, ZO-1, Intracellular signaling, Extracellular signal-regulated kinase, Leaky gut, Microbiology, Gastroenterology

## Abstract

Heat-killed *Lactiplantibacillus plantarum* L-137 (HK L-137) has been suggested to enhance the intestinal barrier in obese mice, leading to improvement of metabolic abnormalities and adipose tissue inflammation, and in healthy humans with overweight, leading to improvement of systemic inflammation. However, its detailed mechanism of action has not been clarified. Therefore, this study investigated the effects of HK L-137 on the permeability of rat small intestinal epithelial IEC-6 cells, tight junction-related gene and protein expression and localization, and intracellular signaling pathways involved in barrier function. Treatment of IEC-6 cells with HK L-137 for 26 h significantly reduced the permeability to fluorescein isothiocyanate-dextran (FD-4). HK L-137 also increased gene and protein expression of zonula occludens-1 (ZO-1), an important tight junction protein, without affecting the localization. Furthermore, inhibition of the extracellular signal-regulated kinase (ERK)1/2 pathway in IEC-6 cells canceled the HK L-137-related reduction in permeability to FD-4. Phosphorylation of ERK in IEC-6 cells was induced 15 min after the addition of HK L-137. These results suggest that HK L-137 reduces intestinal permeability partly through activating the ERK pathway and increasing expression of the ZO-1 gene and protein. Enhancement of intestinal barrier function with HK L-137 might be effective in preventing and treating leaky gut, for which no specific therapeutic tool has been established.

## Introduction

Leaky gut is a condition in which epithelial cell-to-cell junctions are no longer maintained and intestinal permeability is increased, allowing harmful substances such as bacteria, toxins, and viruses to enter the body^1^. Subsequently, such harmful substances are transported via the bloodstream to various organs, leading to the development or exacerbation of many diseases, including chronic inflammation, obesity, diabetes, cardiovascular disease, Alzheimer's disease, autoimmune diseases, allergies, and infectious diseases^1–6^. Therefore, maintaining and promoting intestinal barrier function is critical to people's health.

Tight junctions (TJs), which physically prevent the entry of foreign substances into the body by mechanically connecting epithelial cells, play an important role in the intestinal barrier^7^. TJ formation involves several proteins, including the transmembrane proteins occludin and claudin-1 and zonula occludens-1 (ZO-1), a cytoplasmic protein that binds to these transmembrane proteins^8^. Studies have reported that promoting TJ function improves intestinal barrier function and may be effective in preventing and treating leaky gut^9,10^.

Leaky gut is thought to be caused by diverse factors, including a high-sugar diet, alcohol, aging, intense exercise, and chronic stress^11–13^. Thus, many people are constantly exposed to factors that induce leaky gut in their daily lives. However, currently no established treatment is available for the condition, so early treatment and prevention methods are urgently needed^14^.

Lactic acid bacteria are widely known to improve the intestinal environment and lipid and sugar metabolism and to reduce the risk of developing hypertension, and their health effects have attracted much attention^15–17^. Recently, numerous reports have shown that lactic acid bacteria enhance TJ expression, reduce inflammation, and protect the integrity of the intestinal barrier, raising expectations that lactic acid bacteria can improve intestinal barrier function^18–20^.

*Lactiplantibacillus plantarum* L-137 is a strain isolated from a popular fermented Southeast Asian dish made from fish and rice, and heat-killed *Lactiplantibacillus plantarum* L-137 (HK L-137) has been reported to have many health benefits, including anti-allergy, anti-virus, and anti-tumor effects^21–23^. Clinical studies have shown that because of its immunomodulatory effects, daily consumption of HK L-137 improves health-related quality of life and reduces the incidence of upper respiratory tract infections in participants with high levels of stress^24,25^. Anti-inflammatory effects of HK L-137 have also been reported. For example, HK L-137 promoted colitis recovery in mice treated with dextran sodium sulfate and reduced chronic inflammation in DahlS.Z-*Lepr*^*fa*^/*Lepr*^*fa*^ (DS/obese) rats, a model of metabolic syndrome^26,27^. In addition, HK L-137 has been suggested to enhance intestinal barrier function. For example, it was shown to decrease the permeability of the rainbow trout (Oncorhynchus mykiss) intestinal epithelial cell line (RTgutGC), induce intestinal cell growth by activating gut function in broiler chickens, improve intestinal permeability in obese mouse models of metabolic abnormalities and inflammation, and ameliorate systemic and hepatic inflammation through restoration of the intestinal barrier in humans with overweight^28–31^. However, the mechanism by which HK L-137 improves intestinal barrier function is still unclear.

Intestinal permeability and TJ protein expression have been reported to be regulated by intracellular signaling pathways such as the mitogen-activated protein kinase (MAPK) cascade^32^. Previous studies on lactic acid bacteria showed that extracellular signal-regulated kinase (ERK)1/2, one of the representative pathways of the MAPK family, mediates intestinal barrier function. For example, U0126, a specific inhibitor of MEK-1/2, an upstream kinase that activates ERK1/2, abrogated the protective effect of soluble proteins produced by *Lactobacillus rhamnosus* GG (p40 and p75) against H_2_O_2_-induced tight junction destruction^33^. The inhibition of tumor necrosis factor (TNF)-α-induced downregulation of TJ protein expression by VSL#3, a mixture of eight different strains of probiotics at high concentrations, was also inhibited by the p38 MAPK inhibitors SB203580 and U0126^34^. Furthermore, U0126 attenuated the protective effect of *Lactobacillus salivarius*, which induces phosphorylation of ERK, against TNF-α-induced barrier disruption^35^. Last, the protective effect of *Lactobacillus rhamnosus* GG against interferon-γ- and TNF-α-induced intestinal barrier disruption was abolished by the addition of the MEK-1/2-specific inhibitor PD-98059^36^. Thus, lactic acid bacteria have been found to strengthen the intestinal barrier by regulating intracellular signaling.

Despite the above findings, it is not yet clear whether HK L-137 is involved in the regulation of signaling pathways in the intestinal epithelium. Therefore, in this study, we evaluated the effects of HK L-137 on intestinal barrier function by studying rat small intestinal epithelial IEC-6 cells. We attempted to elucidate the mechanism by which HK L-137 improves intestinal barrier function by evaluating its effect on the expression and localization of genes and proteins related to TJ, and on five signal transduction pathways involved in barrier function.

## Methods

### Preparation of HK L-137

We used a commercial preparation of HK L-137 (House Wellness Foods Corporation, Hyogo, Japan) that contains 20% HK L-137 and 80% dextrin; the HK L-137 in the preparation was prepared as described previously^21^.

### Chemicals

Chemicals were purchased from various suppliers, as follows: Dulbecco’s modified Eagle’s medium (DMEM), penicillin–streptomycin, Bovine insulin, fluorescein isothiocyanate-dextran (FD-4), complete protease inhibitor cocktail tablets, and PhosStop phosphatase inhibitor tablets, from Sigma-Aldrich (Saint Louis, MO, USA); sodium hydrogen carbonate, radioimmunoprecipitation assay (RIPA) buffer, PD98059, dorsomorphin, paraformaldehyde, polyoxyethylene (10) octylphenyl ether (TritonX-100), from Fujifilm Wako (Osaka, Japan); fetal bovine serum (FBS), from HyClone (Logan, UT, USA); primary anti-mouse antibodies for Akt (pan), primary anti-rabbit antibodies for phospho-Akt (Ser473), phospho-p44/42 MAPK (Erk1/2) (Thr202/Tyr204), p44/42 MAPK (Erk1/2), LY294002, SP600125, SB203580, from Cell Signaling Technology (Danvers, MA, USA); anti-mouse occludin antibody, anti-rabbit ZO-1 antibody, donkey anti-rabbit IgG (H + L) highly cross-adsorbed secondary antibody-alexa fluor 488 (secondary fluorescent dye-conjugated donkey anti-rabbit IgG), from Invitrogen (Waltham, MA, USA); horseradish peroxidase (HRP)-conjugated anti-mouse secondary antibody, HRP-conjugated anti-rabbit secondary antibody from Protein Simple (San Jose, CA, USA); Cellstain^®^-DAPI, from DOJINDO LABORATORIES (Kumamoto, Japan); anti-rabbit ZO-1 antibody, from Proteintech Group (Rosemont, IL, USA); FluoroQuest™ Anti-fading Kit II (antifade agent), from AAT Bioquest (Pleasanton, CA, USA); normal donkey serum, from Jackson Immuno Research Laboratories (West Grove, PA, USA); and phosphate buffered saline (PBS) tablets, from Takara (Shiga, Japan).

### Cell culture

IEC-6 cells, a rat small intestine epithelial cell line (European Collection of Authenticated Cell Cultures, Salisbury, UK) that is widely used as a small intestinal epithelial model, were maintained in DMEM containing 10% FBS, 4 μg/mL insulin, 100 U/mL penicillin, and 100 µg/mL streptomycin at 37 °C under an atmosphere of 5% CO_2_. In the following experiments, HK L-137 was added 3 days after seeding, when IEC-6 cells reached 80–90% confluence.

### Effect of HK L-137 on barrier function of IEC-6 cells

We evaluated the barrier function of IEC-6 cells by assessing FD-4 permeability. For the assays, IEC-6 cells were seeded onto TC 24-well inserts (Sarstedt, Nümbrecht, Germany) at 1.8 × 10^4^ cells per well in 0.2 mL of DMEM containing 10%FBS, 4 µg/mL insulin, 100 U/mL penicillin, and 100 µg/mL streptomycin. After 3 days of culture, HK L-137 (500 µg/mL) was added to the apical side of the cells. FD-4 (0.1 mg/mL) was added to the apical side 26 h later, and the fluorescence intensity (excitation, 490 nm; emission, 520 nm) of the FD-4 transmitted to the basolateral side of the cells was measured 5 h later with a NIVO 3S plate reader (PerkinElmer, Waltham, MA, USA). The amount of FD-4 in the sample was calculated by using the calibration curve.

### Expression of TJ-related genes

We evaluated the expression of TJ-related genes by reverse transcription polymerase chain reaction (RT-PCR). First, IEC-6 cells were seeded onto 24-well plates at 1.0 × 10^5^ cells per well in 1 mL of DMEM containing 10% FBS, 4 µg/mL insulin, 100 U/mL penicillin, and 100 µg/mL streptomycin. After culturing for 3 days, cells were treated with HK L-137 (500 μg/mL) for 9 h. Then, total RNA was collected with a Maxwell^®^ RSC simplyRNA Cells Kit (Promega, Madison, WI, USA) and automated RNA extraction system by using the Maxwell^®^ RSC Instrument (Promega) according to the manufacturer’s instructions^37–39^. The RNA was eluted with 50 μL of nuclease-free water, and RNA was quantified with a Nanodrop OneC (Thermo Fisher Scientific, Madison, WI, USA). Then, expression of ZO-1, occludin, claudin-1, claudin-2, and HPRT1 (internal standard) genes was assessed by RT-PCR^40^. Complementary DNA synthesis and RT-PCR were performed with a Thermal Cycler Dice Real Time System TP970 (Takara) and One Step TB Green^®^ PrimeScript™ RT-PCR Kit II (Takara), respectively, according to the manufacturer’s protocol. RT-PCR primers were obtained from FASMAC (Kanagawa, Japan); the primer sequences are shown in Supplemental Table. Data processing was performed with the 2^−ΔΔCT^ method^41^, which was based on analysis of the second derivative curve of amplified plots and performed with Thermal Cycler Dice Real Time System software (version 6.01C, Takara). Target gene expression was normalized for expression of HPRT1 mRNA, which was confirmed to be stable by preliminary analysis.

### Expression of TJ-related proteins

#### Preparation and measurement of proteins

IEC-6 cells were seeded onto 6-well plates at 5 × 10^5^ cells per well in 3 mL of DMEM containing 10% FBS, 4 µg/mL insulin, 100 U/mL penicillin, and 100 µg/mL streptomycin. After 3 days of culture, HK L-137 (500 μg/mL) was added. After 24 h, the supernatant was removed, and the wells were washed with ice-cold PBS. After removal of PBS, ice-cold RIPA buffer supplemented with protease inhibitor cocktail tablets and phosphatase inhibitor tablets was added, and the cells were collected with a cell scraper (Techno Plastic Products AG, Trasadingen, Switzerland). After 1 h on ice, the extract solution was centrifuged at 16,260*g* for 30 min at 4 °C, and the supernatant was collected. Total protein levels in the preparations were determined with a Micro Bicinchoninic Acid (BCA) Protein Assay kit (Thermo Fisher Scientific, Rockford, IL, USA).

#### Western blot analysis

We used Western blot analysis to examine the expression of TJ-related proteins in IEC-6 cells. Automated capillary electrophoresis-based Western blot analyses were performed on the ProteinSimple Wes^®^ System with a 12–230 kDa Separation Module kit (ProteinSimple SM-W004), Total Protein Detection Modulekit (ProteinSimple DM-TP001), the Anti-Rabbit Detection Module kit (ProteinSimple DM-001), and the Anti-Mouse Detection Module kit (ProteinSimple DM-002) according to the manufacturer’s instructions^42–44^. In brief, samples were diluted to an appropriate concentration (0.5 mg/mL) in 0.1 × sample buffer containing sodium dodecyl sulfate, then combined with 5× Fluorescent Master Mix (containing 5× sample buffer, 5× fluorescent standard, and 200 mM dithiothreitol) at a ratio of 4:1 and heated at 95 °C for 5 min. The samples, blocking reagent (antibody diluent), primary antibodies, HRP-conjugated secondary antibodies, and chemiluminescent substrate (luminol-S/peroxide) were added to each well of the microplate provided by the manufacturer. The microplate was loaded into the instrument, which performed electrophoretic protein separation and immunodetection in the automated capillary system. Expression of total protein was used as the internal control for ZO-1 or occludin after 24-h culture. The data were analyzed with Compass software (version 4.1.0; ProteinSimple).

### Effect of HK L-137 on ZO-1 localization

We performed immunofluorescence staining to investigate the effect of HK L-137 on localization of the TJ protein ZO-1. IEC-6 cells were seeded onto a μ-Slide 8 Well (NIPPON Genetics, Tokyo, Japan) at 1.2 × 10^4^ cells per well in 0.3 mL of DMEM containing 10% FBS, 4 µg/mL insulin, 100 U/mL penicillin, and 100 µg/mL streptomycin. After culturing for 3 days, the cells were treated with HK L-137 (500 μg/mL) for 24 h and then fixed with 4% paraformaldehyde. After blocking with PBS containing 0.3% TritonX-100 and 0.6% normal donkey serum (blocking buffer) and adding the primary anti-ZO-1 antibody, the slide was incubated at 4 °C overnight. Then, it was incubated with the secondary fluorescent dye-conjugated donkey anti-rabbit IgG for 1 h in the dark. After adding DAPI and incubating for 10 min to stain the nucleus, antifade agent was added; ZO-1 was stained green, and nuclei were stained blue. Between each step, each well was washed three times with PBS containing 0.1% TritonX-100. ZO-1 localization was imaged (excitation, 490 nm; emission, 525 nm; ZO-1, 360 nm/460 nm: nucleus) by a confocal laser scanning microscope AX R (Nikon, Tokyo, Japan).

### Effect of specific inhibitors of intracellular signaling pathways on the HK L-137-related reduction in permeability to FD-4 in IEC-6 cells

We focused on five pathways (ERK, c-Jun N-terminal kinases [JNK], p38, AMP-activated protein kinase [AMPK], and Akt) that have been reported to be particularly relevant to epithelial barrier function. To investigate whether these signaling pathways are involved in the HK L-137-induced reduction in permeability, we performed an FD-4 permeability assay with specific inhibitors of ERK1/2 (PD98059), JNK (SP600125), p38 (SB203580), AMPK (dorsomorphin), and Akt (LY294002). For the assays, HK L-137 was added to the apical side of the cells pretreated with each inhibitor for 1 h. Inhibitor concentrations were determined based on previous studies, and treatment times were aligned to 1 h^45–48^. Culture of IEC-6 cells and measurement of FD-4 were performed as described above.

### Effect of HK L-137 on phosphorylation levels of intracellular signaling pathways in IEC-6 cells

We assessed whether HK L-137 activates the ERK, AMPK, and Akt pathways by treating IEC-6 cells with HK L-137 for 15 min and measuring phosphorylation levels. We also investigated whether pathway activation by HK L-137 was suppressed by pre-treating cells for 1 h with a pathway inhibitor, such as PD98059, dorsomorphin, or LY294002, and then applying HK L-137 (500 μg/mL) for 15 min. IEC-6 cell culture and Western blot analysis were performed as described above. Phosphorylated ERK, Akt, or AMPK expression after 15-min culture was expressed as the ratio of phosphorylated to total ERK, Akt, or AMPK protein.

### Statistical analysis

Control and treated cells were compared with Student’s *t* test or Newman–Keuls test. Results are shown as the mean and standard deviation (SD). All treatments were biologically replicated at least three times. A probability (P) value of less than 0.05 was defined as indicating statistical significance, and analyses were performed with GraphPad Prism6 software (GraphPad, CA, USA).

## Results

### HK L-137 improves the barrier function of IEC-6 cells

**Figure 1:**
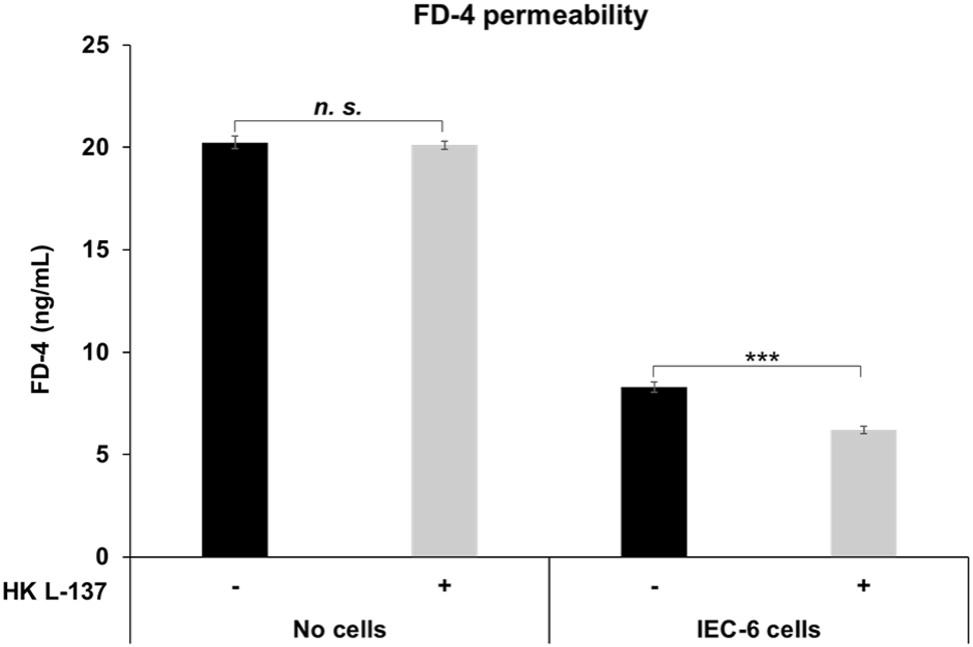
Effect of HK L-137 on FD-4 permeability of IEC-6 cells. FD-4 was added to rat IEC-6 cells treated with HK L-137 (500 μg/mL) for 26 h, and fluorescence intensity was measured 5 h later. Excitation wavelength 490 nm, emission wavelength 520 nm. The figure shows the beneficial effect of HK L-137 on intestinal barrier function. Means ± SD, *n* = 4, Newman–Keuls test, ***P < 0.001. *FD-4* fluorescein isothiocyanate-dextran, *HK L-137* heat-killed *Lactiplantibacillus plantarum* L-137.

In the absence of IEC-6 cells, the addition of HK L-137 had no effect on FD-4 permeability, but treatment of IEC-6 cells with HK L-137 for 26 h resulted in a significant decrease in FD-4 permeability (Fig. 1). This result suggests that HK L-137 acts directly on IEC-6 cells to reduce their permeability.

### Effect of HK L-137 on gene expression in IEC-6 cells

**Figure 2:**
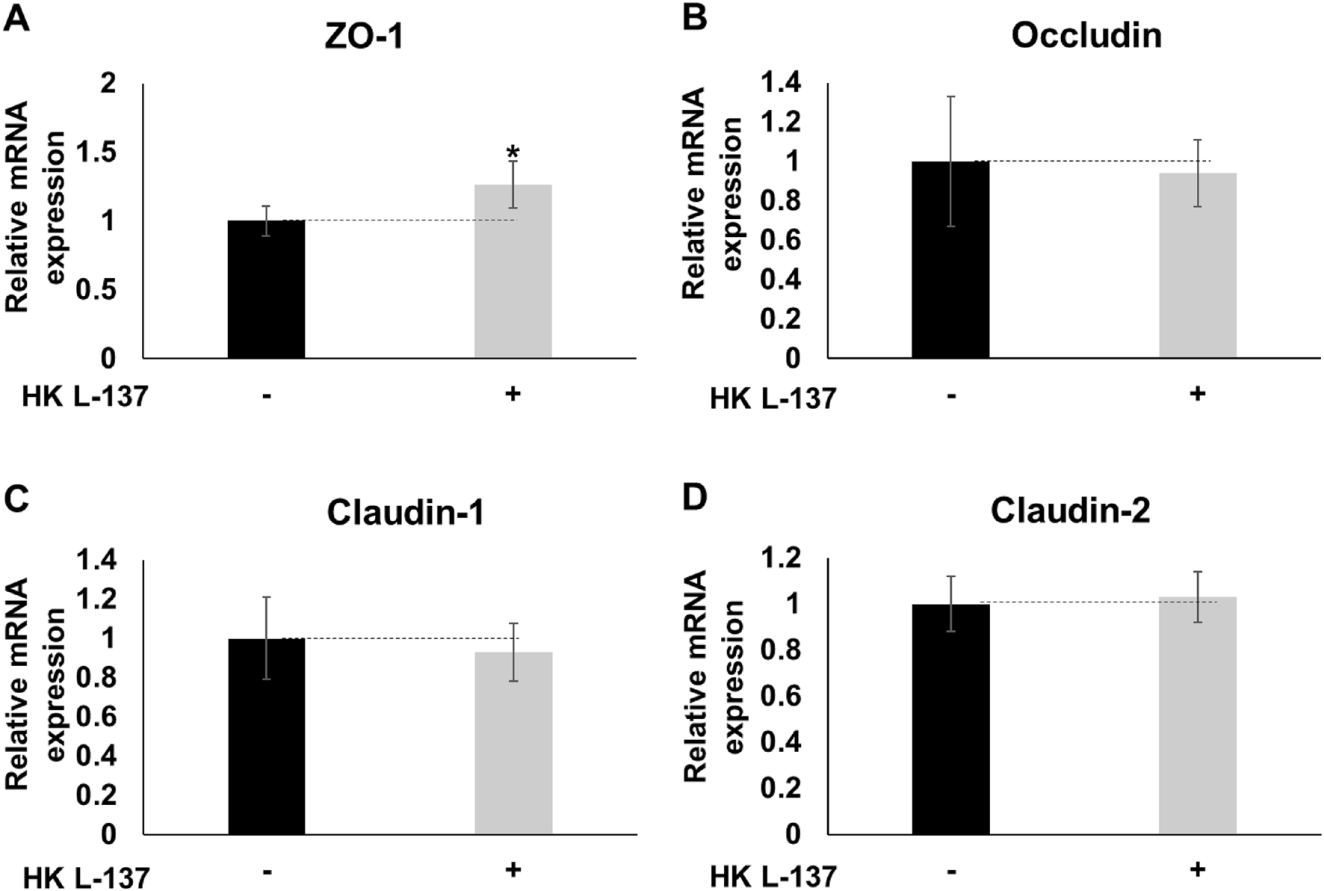
Effect of HK L-137 on expression in IEC-6 cells of tight junction-related genes. Tight junction-related genes in rat IEC-6 cells treated with HK L-137 (500 μg/mL) for 9 h were measured by reverse transcription polymerase chain reaction. (**A**) ZO-1, (**B**) occludin, (**C**) claudin-1, (**D**) claudin-2. The expression level of each gene was corrected by using the Hrpt1 gene expression level as an internal standard. Means ± SD; *n* = 4; Student’s *t* test, *P < 0.05 (vs. cells untreated with HK L-137). *HK L-137* heat-killed *Lactiplantibacillus plantarum* L-137, *ZO-1* zonula occludens-1.

Treatment of IEC-6 cells with HK L-137 for 9 h had no effect on the expression of the TJ genes occludin, claudin-1, and claudin-2, but it did increase ZO-1 gene expression (Fig. 2A–D). The increased ZO-1 expression, key regulators of TJ formation, may explain why HK-L-137 decreased the permeability of IEC-6 cells.

### Effect of HK L‑137 on protein expression in IEC-6 cells

**Figure 3:**
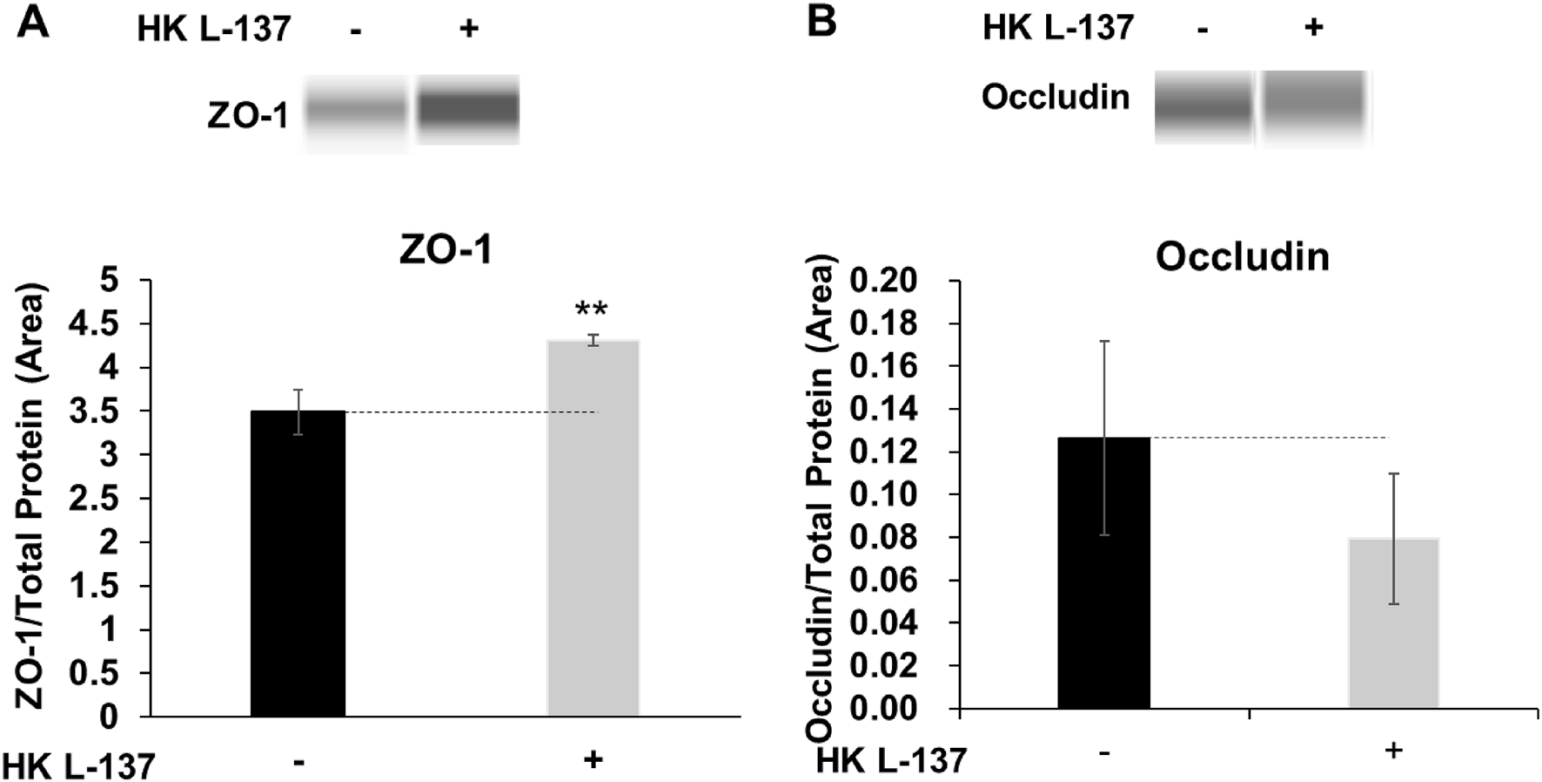
Effect of HK L-137 on tight junction protein expression in IEC-6 cells. Tight junction protein expression in rat IEC-6 cells treated with HK L-137 (500 μg/mL) for 24 h was measured by Western blot analysis. Images of representative bands are shown. Full-length images are shown in Supplemental Fig. S1. ZO-1 (**A**) and occludin (**B**) expression levels were corrected for total protein expression levels. Means ± SD; *n* = 3; Student’s *t* test, **P < 0.01 (vs. cells untreated with HK L-137). *HK L-137* heat-killed *Lactiplantibacillus plantarum* L-137, *ZO-1* zonula occludens-1.

After treatment of IEC-6 cells with HK L-137 for 24 h, the expression level of ZO-1 protein increased significantly (Fig. 3A), but no significant change was seen in the expression level of occludin (Fig. 3B). This increase in ZO-1 protein expression levels by HK L-137 may contribute to the enhancement of barrier function by HK L-137.

### Effect of HK L-137 on ZO-1 localization in IEC-6 cells

**Figure 4:**
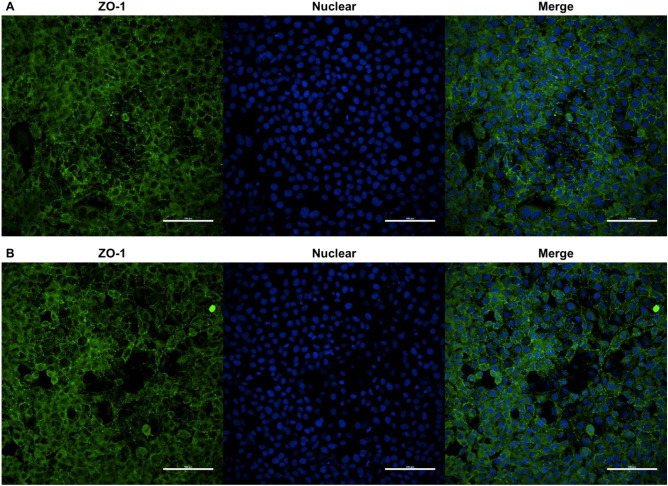
Effect of HK L-137 on ZO-1 localization in IEC-6 cells by immunofluorescence staining. ZO-1 expression in rat IEC-6 cells at 24 h without (**A**) or with (**B**) HK L-137 (500 μg/mL) was visualized and analyzed with a Confocal Laser Scanning Microscope. ZO-1 was stained green, and nuclei were stained blue (scale bar = 100 μm). Independent treatment was carried out in triplicate and representative images are shown. *ZO-1* zonula occludens-1.

Immunofluorescence staining to investigate the effect of HK L-137 on ZO-1 localization showed no change between untreated and HK L-137-treated cells, and ZO-1 was correctly localized at the intercellular junctions in both conditions (Fig. 4A,B). Thus, HK L-137 does not appear to decrease permeability by affecting cell density or ZO-1 localization.

### The ERK pathway is involved in HK L-137-induced barrier function improvement in IEC-6 cells

**Figure 5:**
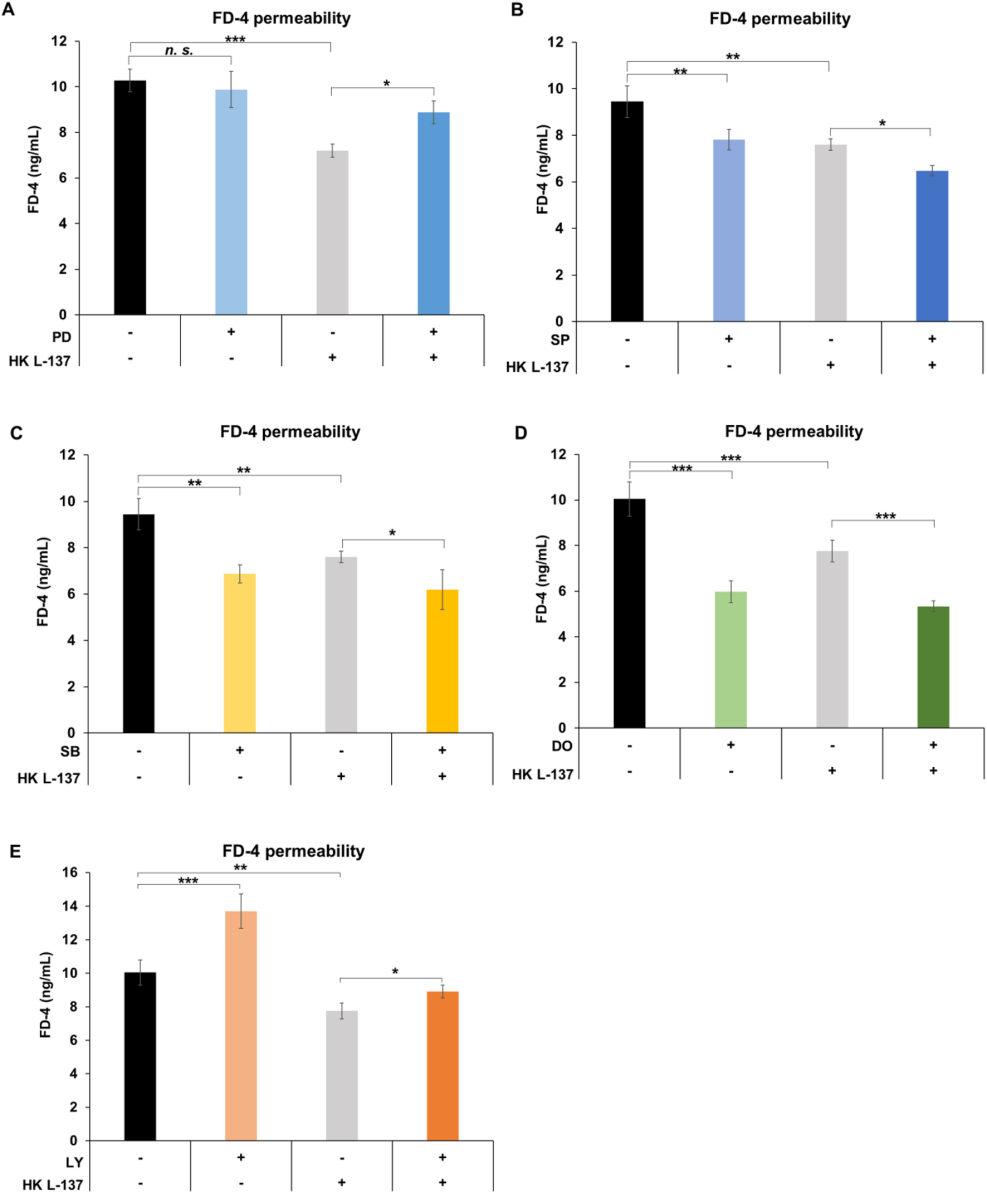
Effect of specific pathway inhibitors (ERK1/2; PD98059, JNK; SP600125, p38; SB203580, AMPK; dorsomorphin, Akt; LY294002) on improving FD-4 permeability of IEC-6 cells by HK L-137. FD-4 was added to rat IEC-6 cells treated with (**A**) PD98059 (25 μM), (**B**) SP600125 (10 μM), (**C**) SB203580 (10 μM), (**D**) dorsomorphin (20 μM), and (**E**) LY294002 (5 μM) for 1 h and then with HK L-137 (500 μg/mL) for 26 h, and fluorescence intensity was measured 5 h later. Excitation wavelength, 490 nm; emission wavelength, 520 nm. Means ± SD; *n* = 3 (**B**,**C**) or 4 (**A**,**D**,**E**), Newman–Keuls test, *P < 0.05, **P < 0.01, ***P < 0.001. *DO* dorsomorphin, *FD-4* fluorescein isothiocyanate-dextran, *HK* *L-137* heat-killed *Lactiplantibacillus plantarum* L-137, *LY* LY294002, *PD* PD98059, *SP* SP600125, *SB* SB203580.

Results of the FD-4 permeability assays showed that treatment of IEC-6 cells with the ERK1/2 inhibitor PD98059 counteracted the HK L-137-related reduction in FD-4 permeability, despite having no effect in the single treatment (Fig. 5A). In contrast, treatment with the JNK inhibitor SP600125, p38 inhibitor SB203580, and AMPK inhibitor dorsomorphin enhanced the reduced permeability by HK L-137, similar to the effect of single treatment (Fig. 5B–D). These results suggest that the JNK, p38, and AMPK pathways have distinct roles in the intestinal barrier that are not affected by HK-L-137. On the other hand, the Akt inhibitor LY294002, which increased the permeability in the single treatment, also increased the permeability of IEC-6 cells treated with HK L-137 (Fig. 5E). Therefore, the Akt inhibitor appears to have counteracted the action of HK L-137 through a different pathway. These results suggest that the ERK pathway is involved in the reduced FD-4 permeability of IEC-6 cells treated with HK L-137.

### Effect of HK L-137 on phosphorylation levels of intracellular signaling pathways in IEC-6 cells

**Figure 6:**
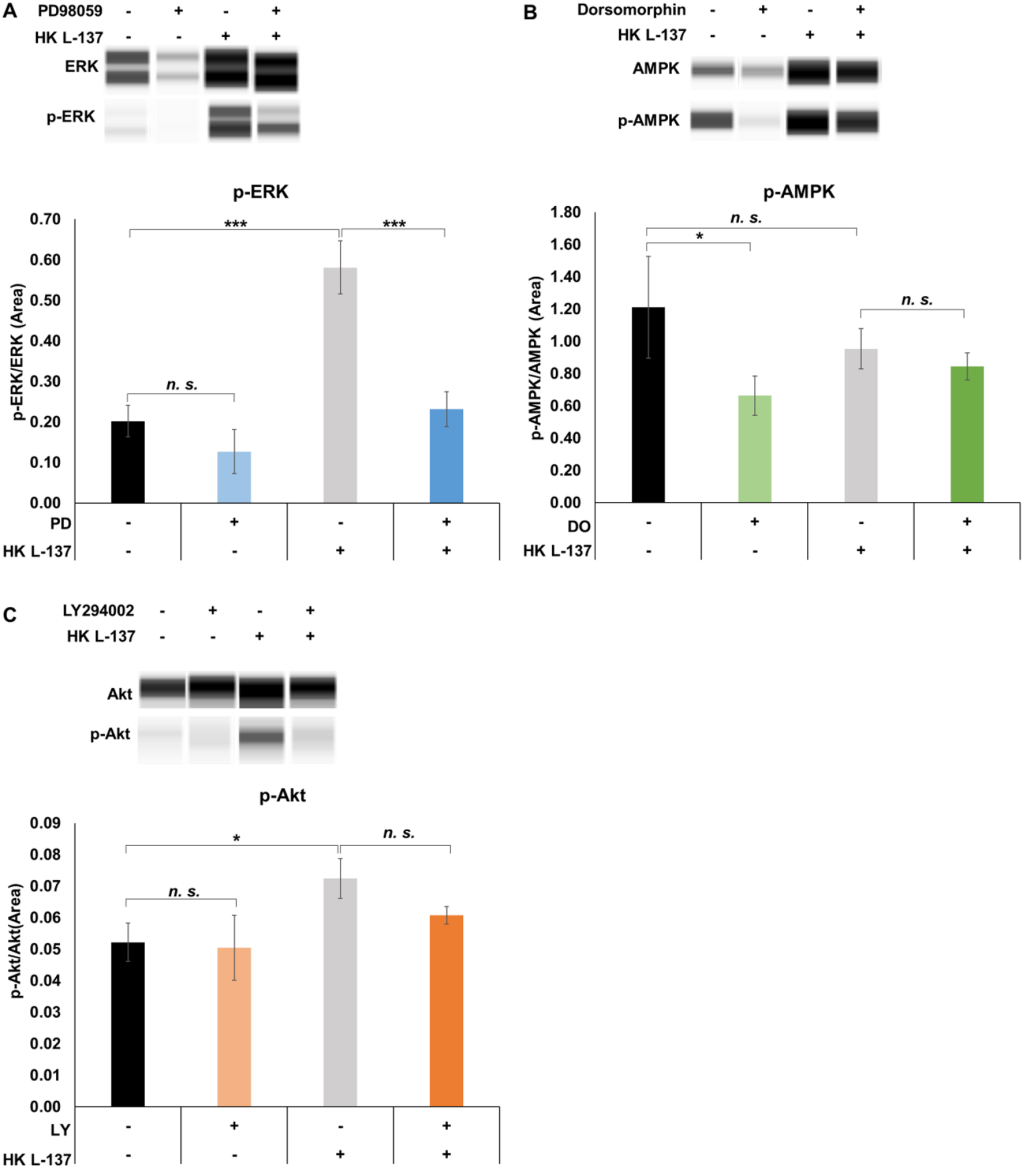
Effect of HK L-137 on phosphorylated protein expression levels in IEC-6 cells. (**A**) p-ERK/ERK, (**B**) p-AMPK/AMPK, (**C**) p-Akt/Akt levels were measured in rat IEC-6 cells treated with or without the inhibitors of ERK1/2 (PD98059, 25 μM), AMPK (dorsomorphin, 20 μM), or Akt (LY294002, 5 μM) for 1 h, followed by 15 min of treatment with HK L-137 (500 μg/mL). Images of representative bands are shown. Full-length images are shown in Supplemental Fig. S2. P-ERK, p-AMPK, and p-Akt expression levels were corrected for ERK, AMPK, and Akt expression levels. The expression levels of ERK and p-ERK were calculated by adding the area values of 42 kDa and 44 kDa. Means ± SD; *n* = 3, Newman–Keuls test, **P < 0.01, ***P < 0.001. *AMPK* AMP-activated protein kinase, *DO* dorsomorphin, *ERK* extracellular signal-regulated kinase, *HK L-137* heat-killed *Lactiplantibacillus plantarum* L-137, *LY* LY294002, *PD* PD98059, *p-AMPK* phosphorylated AMP-activated protein kinase, *p-ERK* phosphorylated extracellular signal-regulated kinase.

Treatment of IEC-6 cells with HK L-137 for 15 min led to a significant increase in phosphorylation levels of ERK1/2, and prior treatment with the ERK1/2 inhibitor PD98059 for 1 h significantly suppressed the increase in phosphorylation levels (Fig. 6A). In contrast, no change was observed in the phosphorylation level of AMPK after HK L-137 treatment (Fig. 6B). However, similar to ERK, the phosphorylation level of Akt was significantly increased by HK L-137 treatment, whereas prior treatment with the Akt inhibitor LY294002 had no significant effect on the HK L-137-related increase in phosphorylation levels (Fig. 6C). Therefore, we concluded that HK L-137 activates the ERK pathway directly and the Akt pathways by a route that remains to be determined.

## Discussion

HK L-137 reduces intestinal epithelial permeability and may enhance intestinal barrier function, but the mechanism of action is largely unknown^28,30,31^. In this study, by using rat small intestinal epithelial IEC-6 cells, we showed for the first time that HK L-137 decreases permeability by activating the ERK pathway and increases expression of TJ genes and proteins. Because effective treatments for increased intestinal permeability have not been established, these results provide new strategies for the prevention and treatment of leaky gut^14^.

An intact intestinal barrier is known to be important in the prevention of disease and maintenance of health^49^. Rat intestinal epithelial IEC-6 cells are widely used to assess intestinal barrier function in vitro^50^. We found that HK L-137 reduced FD-4 permeability in IEC-6 cells, which may lead to improved barrier function in vivo (Fig. 1). Epithelial cells transport molecules via either transcellular or paracellular routes^51^. The latter comprises two distinct pathways: the pore pathway, which allows small molecules with a certain charge to pass through, and the leak pathway, which allows very small amounts of macromolecules to pass through regardless of their charge^52^. Of these pathways, FD-4 is thought to pass through the barrier via the leak pathway^53^. Excessive passage of antigenic substances such as macromolecules, bacterial products, and food antigens across the epithelial barrier via the leak pathway has been suggested to underlie a variety of diseases. Furthermore, dysregulation of the leak pathway may be a contributing factor to diseases such as inflammatory bowel disease and celiac disease^54–56^. These findings that HK L-137 had a protective effect in a mouse model of dextran sulfate sodium-induced colitis and that it reduced FD-4 permeability of intestinal epithelial cells in this study suggest that HK L-137 may contribute to the treatment and prevention of various intestinal diseases by regulating the leak pathway^26^. This is also supported by the result that HK L-137 suppressed the increase in FD-4 permeability caused by lipopolysaccharide (LPS), which is known to be one of the causes of leaky gut (Supplemental Fig. S3).

Although other factors such as hydrogen peroxide, cytokines, mechanical stress, chemicals, and pathogenic bacteria have been also reported as causes of leak pathway permeability changes, little is understood about the regulatory mechanisms and properties of the leak pathway^57,58^. On the other hand, TJ proteins are known to be involved in the regulation of the leak pathway^59,60^. ZO-1, a TJ scaffold protein, is considered a regulator of the leak pathway, and ZO-1 depletion was reported to disrupt the leak pathway^9,57^. ZO-1 interacts with the transmembrane proteins claudin and occludin, with cytoskeletal F-actin, and with myosin and other signaling proteins and is essential for TJ and cytoskeletal structure; however, its role is not clearly understood^9^. On the other hand, paracellular permeability was reported to be reduced by upregulation of endogenous ZO-1 expression^61^. In this study, we found that HK L-137 increased ZO-1 gene and protein expression (Figs. 2A and 3A). Increased ZO-1 localization at or near tight junctions was also suggested to contribute to decreased paracellular permeability^61^. On the other hand, accumulating data suggest that ZO-1 delocalized from TJs could be implicated in the regulation of tumor-promoting genes^62^. In the present study, HK L-137 had little effect on the localization of ZO-1 (Fig. 4). Similarly, propolis collected by honeybees from plant sources and PHSRN peptides derived from fibronectin, an adhesive glycoprotein of the extracellular matrix, increase the expression of ZO-1 without affecting its localization^45,63^. In addition to having a structural role in TJ organization, ZO-1 contributes to TJ-independent epithelial repair through Wnt signaling and mitotic spindle orientation. Thus, preventing ZO-1 loss in patients with inflammatory bowel disease may promote mucosal healing^64^. Considering that ZO-1 is critical for upregulation of epithelial proliferation, its activation may have a positive effect on intestinal morphology, thereby improving absorptive ability and promoting growth. Indeed, feeding of HK L-137 to tilapia, snakehead, common carp, broiler chickens, and pigs was reported to improve intestinal morphology and/or growth performance^65–69^. We found that HK L-137 enhances ZO-1 expression without altering its localization, supporting the potential role of ZO-1 in intestinal barrier improvement by HK L-137. The mechanism by which the HK L-137-related increase in ZO-1 contributes to TJ and barrier function requires further study.

ZO-1 expression may be upregulated through signaling pathways such as the ERK and Akt pathways^63,70^. We found that addition of an ERK inhibitor inhibited the permeability-lowering effect of HK L-137 on FD-4 and that HK L-137 increased phosphorylation levels of ERK1/2 (Figs. 5A, 6A). ERK1/2 may increase the transcriptional activity of the transcriptional regulator cyclic AMP response element-binding protein (CREB), leading to increases in target gene ZO-1 expression^71^. Furthermore, lipoteichoic acid (LTA), a major component of the cell wall of gram-positive bacteria, was reported to activate the ERK pathway in macrophages, melanomas, and corneas and to activate ERK through Toll-like receptor 2 (TLR2)^72–75^. In previous studies, we showed that the expression levels of LTA on the cell surfaces is higher in HK L-137 than those in common lactic acid bacteria^76^. Therefore, we suggest that TLR2 in IEC-6 cells recognize LTA, which is abundantly expressed in HK L-137, which activates the ERK pathway and thereby enhances ZO-1 expression via increased transcriptional activity of CREB and contributes to the permeability-lowering effect of HK L-137. Besides mediating an increase in TJ expression, the ERK pathway has been shown to have other effects. This is supported chronologically by the results of this study, in which phosphorylation of ERK occurred 15 min after addition of HK L-137, ZO-1 mRNA expression increased after 9 h, ZO-1 protein expression levels increased after 24 h, and FD-4 permeability decreased after 26 h. For example, activation of ERK in IEC-6 cells by TGF-β1 was reported to accelerate wound closure and epithelial tissue repair via the Ras/MEK/ERK pathway, and activation of the ERK pathway under hyperoxia in human intestinal epithelial cells was found to promote interleukin-17D expression and protect the intestinal barrier^77–79^. The results of the present study indicate that HK L-137 may improve barrier function through the ERK pathway, but the detailed mechanism requires further study.

The Akt inhibitor increased permeability when added without HK L-137, and it inhibited the FD-4 permeability-lowering effect of HK L-137 (Fig. 5E). HK L-137 increased the phosphorylation level of Akt, whereas the Akt inhibitor alone had no effect on the increased phosphorylation levels (Fig. 6C). Intestinal barrier function-enhancing effects via the Akt pathway have been reported; for example, zinc promotes the expression of the TJ protein ZO-1 and improves intestinal barrier function by activating PI3K/AKT/mTOR signaling, and the breast milk factor erythropoietin protects ZO-1 expression and barrier function by activating Akt^80,81^. In this study, HK L-137 appeared to improve barrier function via the Akt pathway, although it is possible that the Akt and ERK pathways may together contribute to the permeability-lowering effect of HK L-137. This study attempted to elucidate the mechanism by which HK L-137 enhanced intestinal barrier function by using rat small intestinal epithelial cells; however, further investigation of the recognition mechanism of HK L-137 and its mechanism of action related to ZO-1 expression and the ERK pathway in the human intestinal epithelium is needed.

## Conclusions

In conclusion, in rat small intestinal IEC-6 cells, HK L-137 was shown to increase ZO-1 expression and decrease FD-4 permeability, possibly through activation of the ERK pathway. Our results provide new insights into the mechanism of the intestinal health-promoting effects of lactic acid bacteria and suggest that HK L-137 may be useful in the prevention and treatment of leaky gut. However, the mechanism of action and downstream pathways of HK L-137 in IEC-6 cells need to be further investigated in future studies.

### Supplementary Information

Supplementary Table 1.

Supplementary Figure S1.

Supplementary Figure S2.

Supplementary Figure S3.

Supplementary Legends.

## Data Availability

The datasets used and/or analysed during the current study are available from the corresponding author on reasonable request.
