# Supplementary material for: Beneficial effect of heat-killed Lactiplantibacillus plantarum L-137 on intestinal barrier function of rat small intestinal epithelial cells
Source: Sci Rep. 2024 May 29;14:12319. doi: 10.1038/s41598-024-62657-0 (PMC11136994; doi:10.1038/s41598-024-62657-0)
Supplement: Supplementary file 1 — Supplementary Table 1. [file 41598_2024_62657_MOESM1_ESM.doc]

**Supplemental table**

**Supplemental Table Real time PCR primers for analysis.**

| Gene | Direction | Primer sequence |
| --- | --- | --- |
| ZO-1 | Forward （5’-> 3’） | CGCCTCTGTCCAACTCTTCTCT |
|  | Reverse （5’-> 3’） | GGTGTGAATCGGTTGTATGCTG |
| Occludin | Forward （5’-> 3’） | CCTCCTTACAGGCCGGATGA |
|  | Reverse （5’-> 3’） | AGCATTGGTCGAACGTGCAT |
| Claudin-1 | Forward （5’-> 3’） | AGGCAACCAGAGCCTTGAT |
|  | Reverse （5’-> 3’） | CATGCACTTCATGCCAATGGTGGA |
| Claudin-2 | Forward （5’-> 3’） | TCGAGAAAGAACAGCTCCGTTT |
|  | Reverse （5’-> 3’） | TTCGCTTGTCTTTTGGCTGC |
| Hprt1 | Forward （5’-> 3’） | GGTTCTGTCATGTCGACCCT |
|  | Reverse （5’-> 3’） | AGCAAGTCTTTCAGTCCTGTCC |
