# Supplementary figures and images for: Beneficial effect of heat-killed Lactiplantibacillus plantarum L-137 on intestinal barrier function of rat small intestinal epithelial cells
Source: Sci Rep. 2024 May 29;14:12319. doi: 10.1038/s41598-024-62657-0 (PMC11136994; doi:10.1038/s41598-024-62657-0)

**A ZO-1**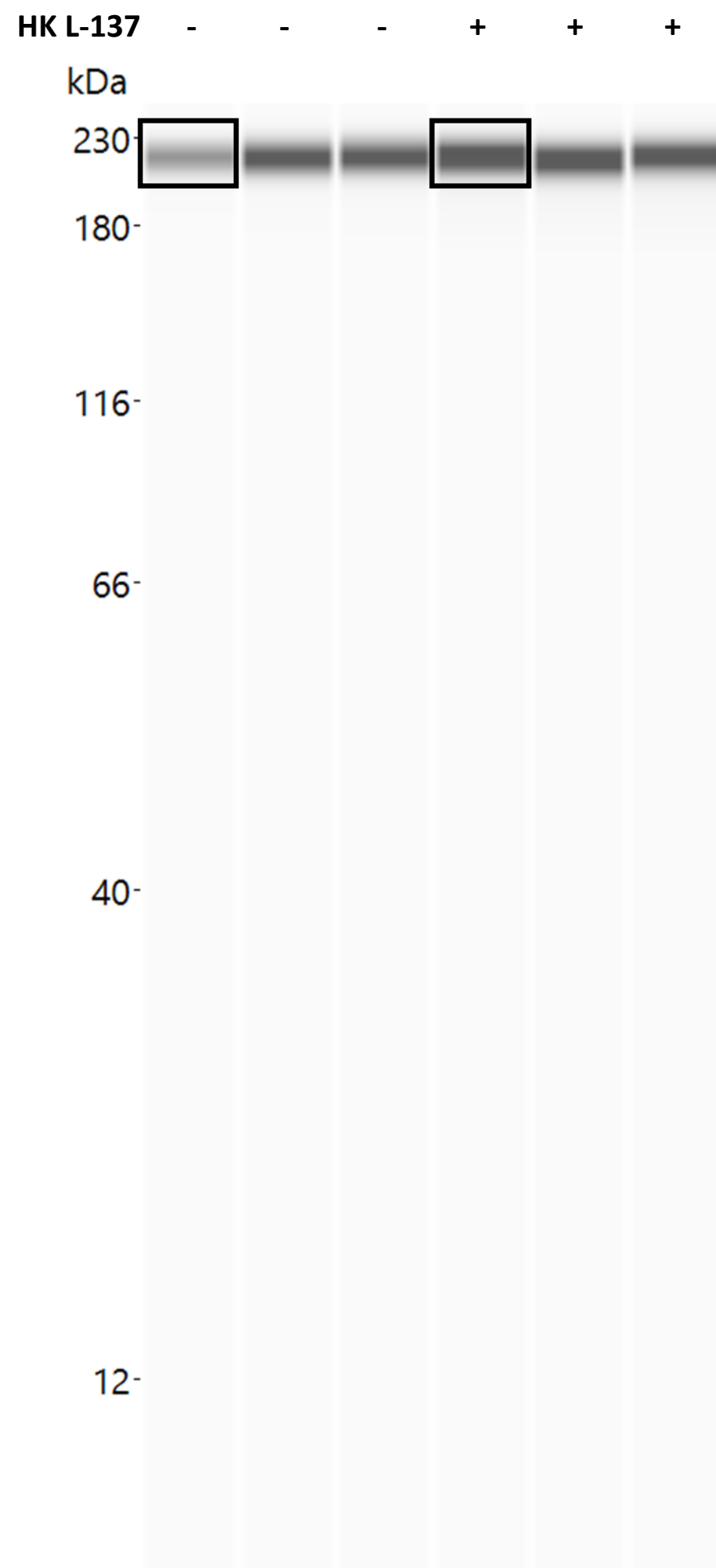**B Occludin**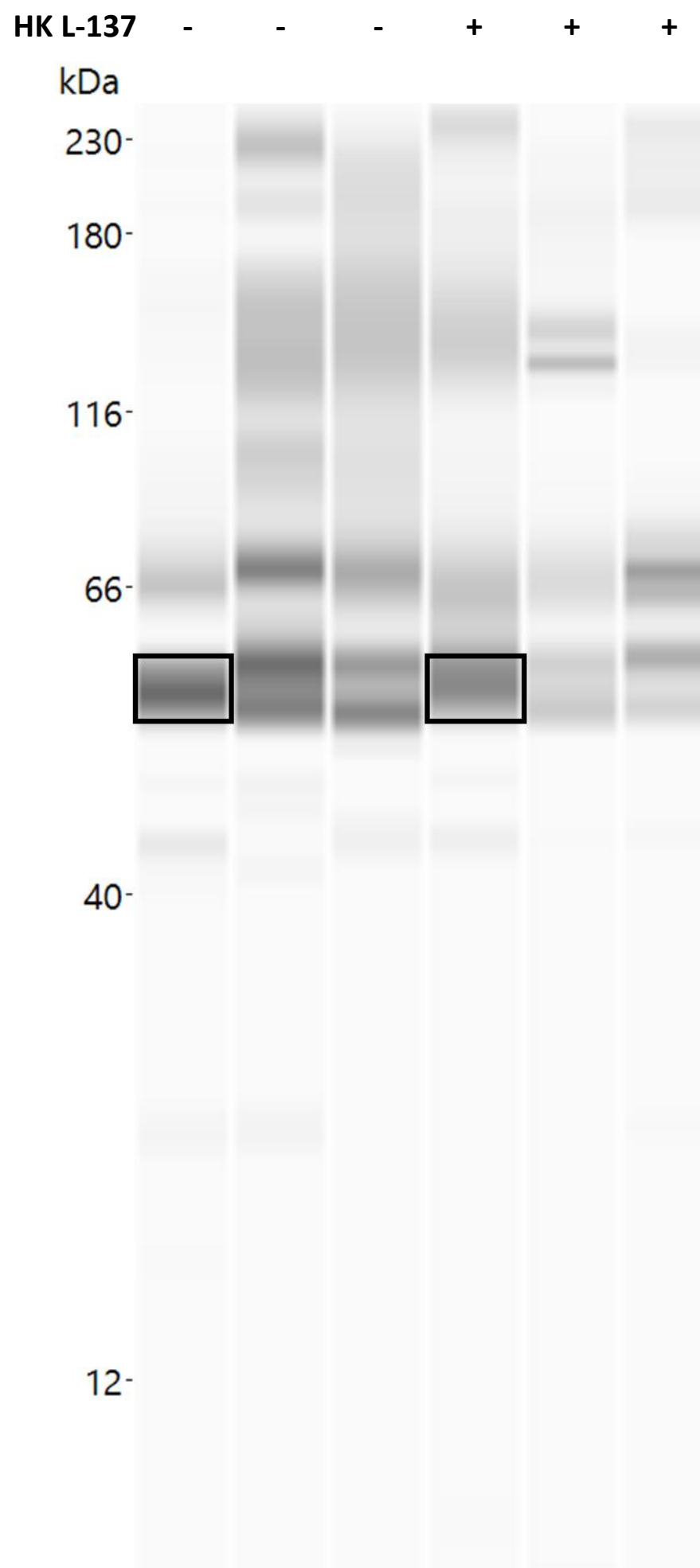**C Total protein**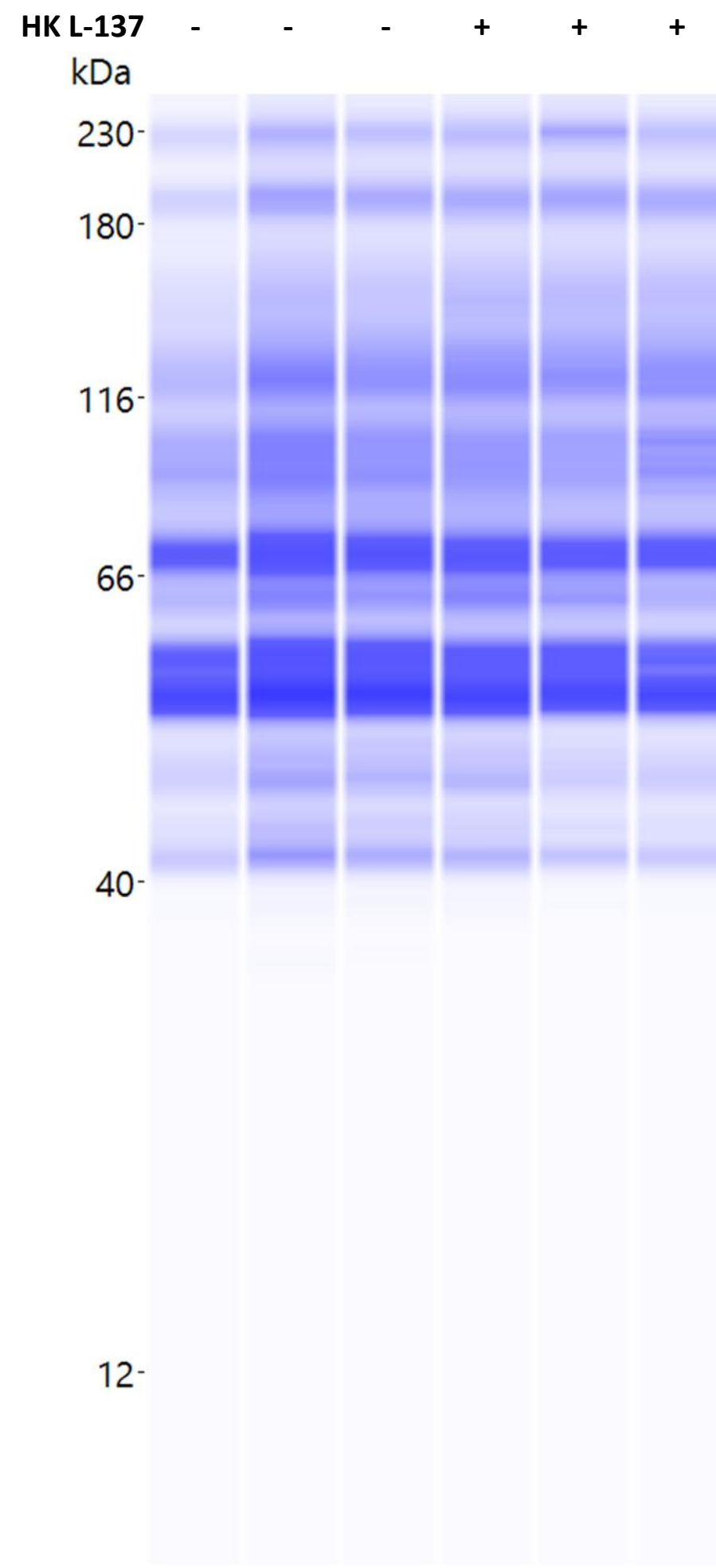

Supplement: Supplementary file 2 — Supplementary Figure S1. [file 41598_2024_62657_MOESM2_ESM.pdf]

A

ERK

p-ERK

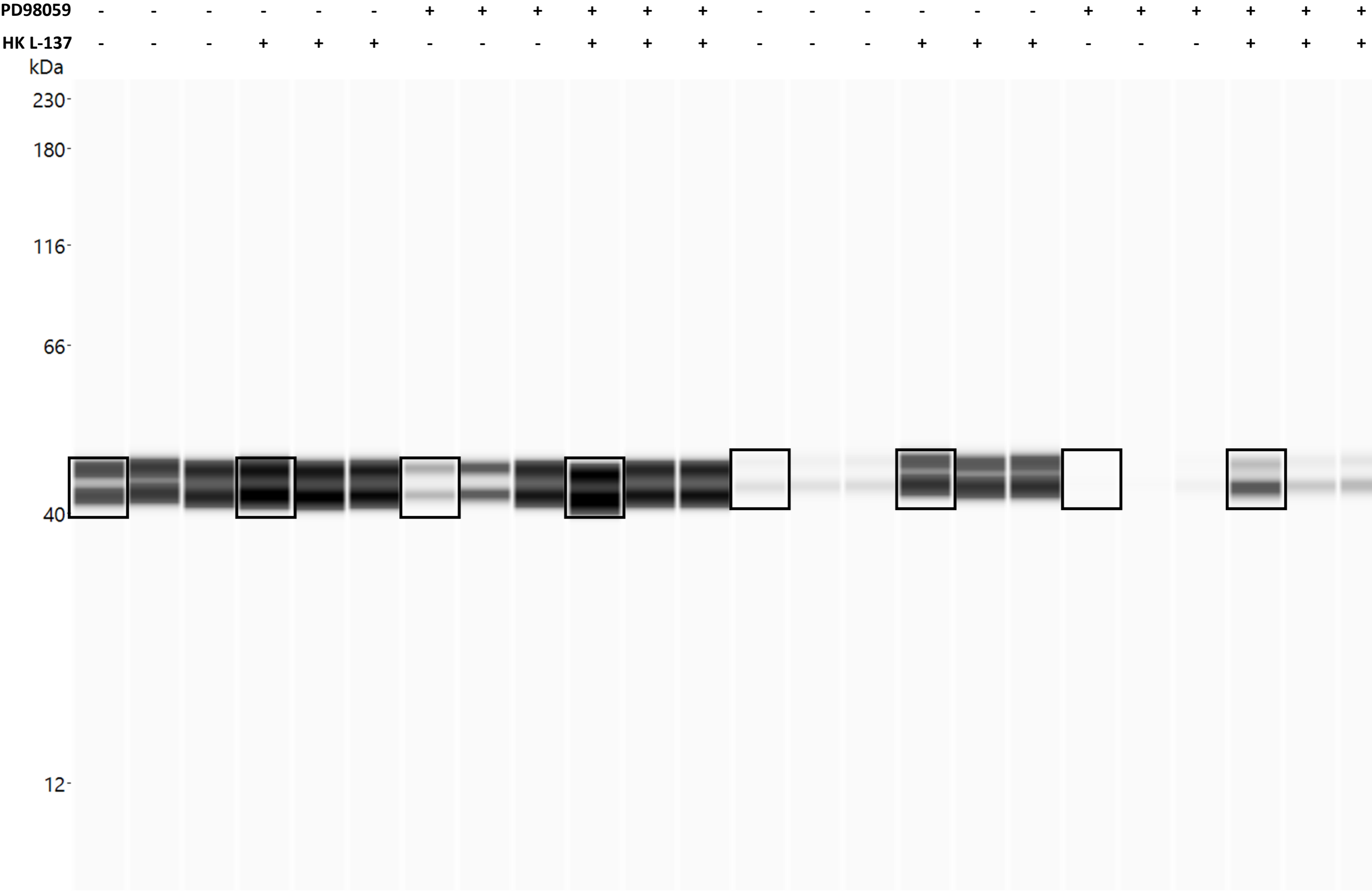

B

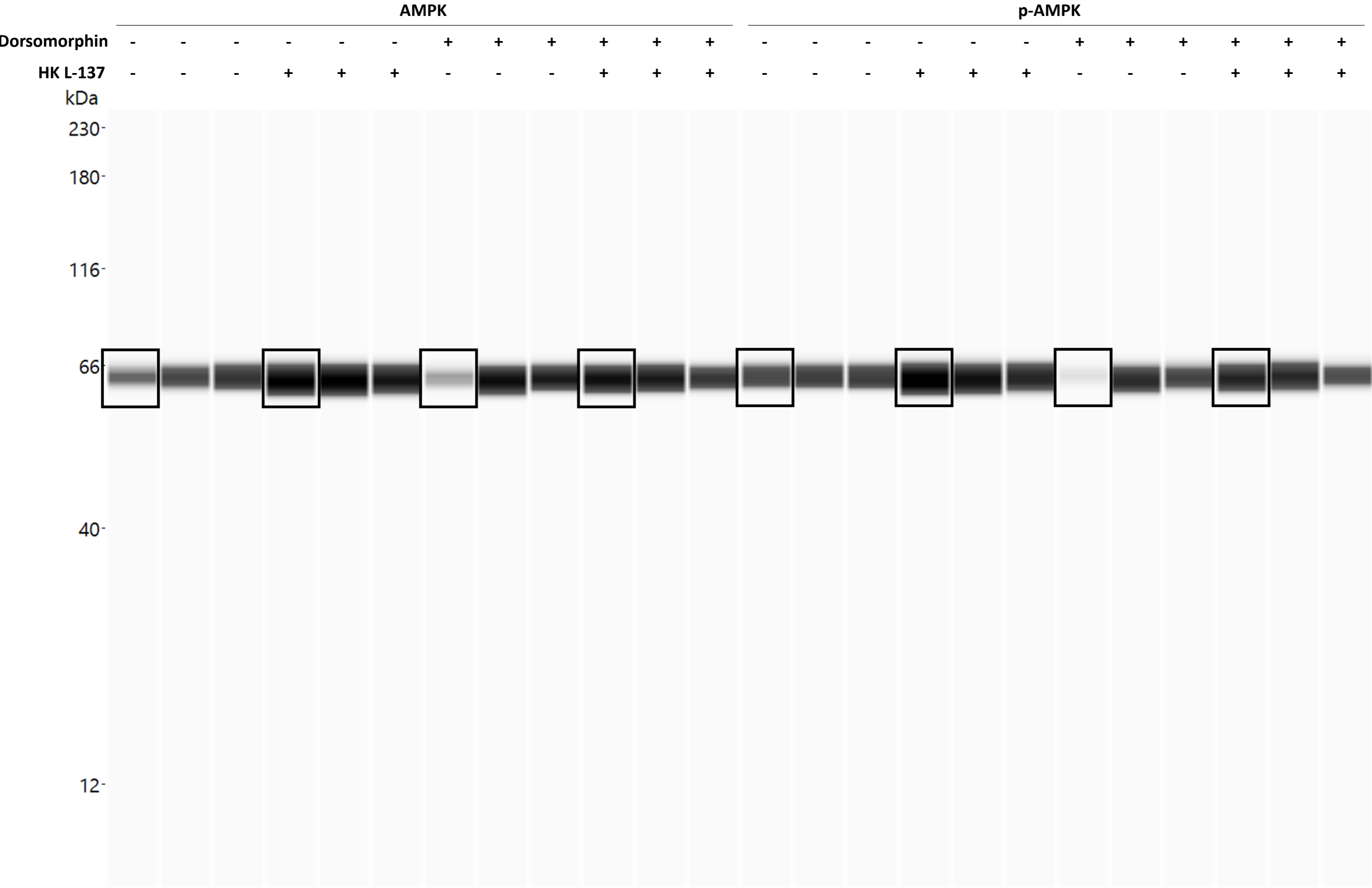

C

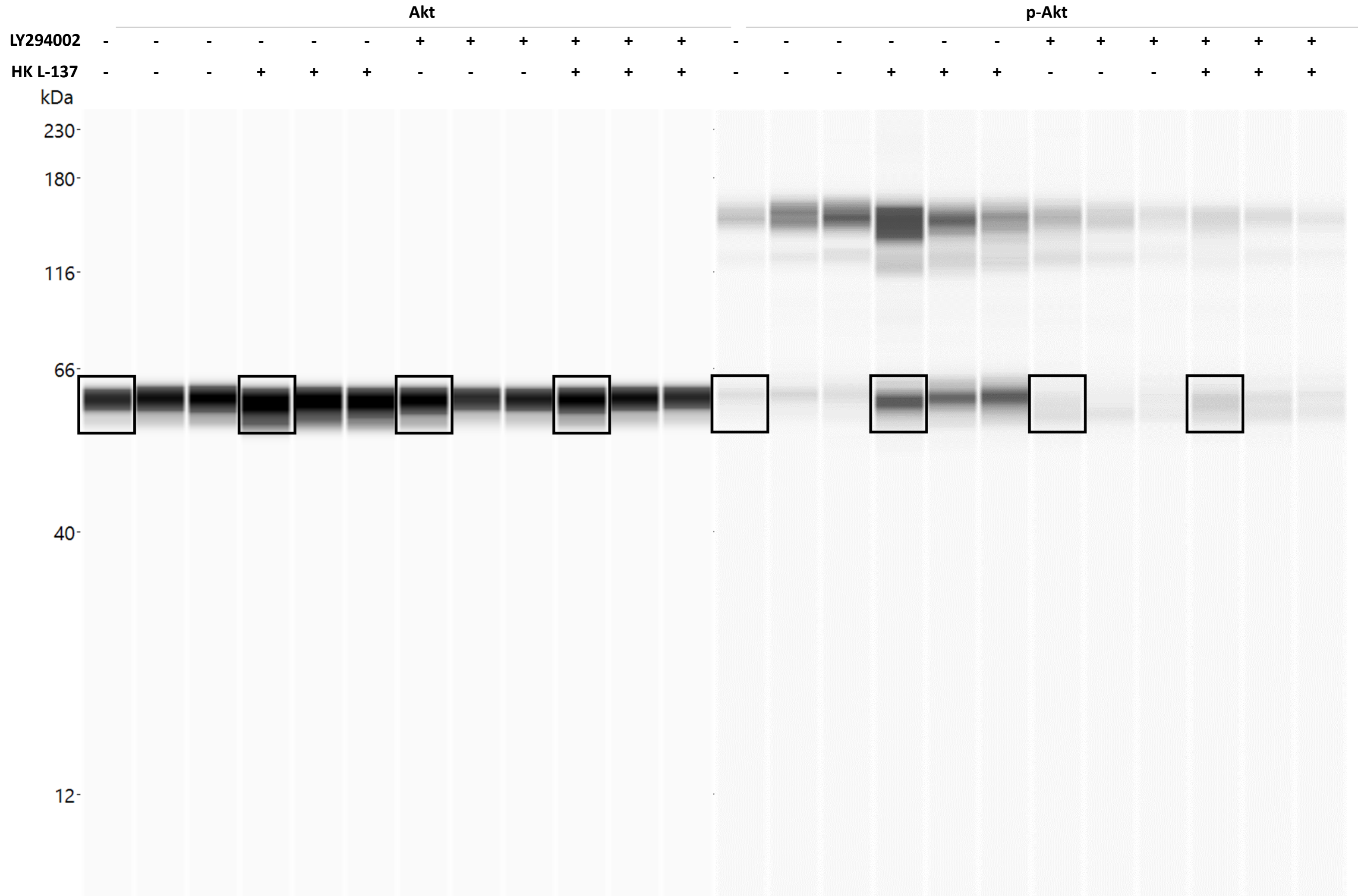

Supplement: Supplementary file 3 — Supplementary Figure S2. [file 41598_2024_62657_MOESM3_ESM.pdf]

# FD-4 permeability

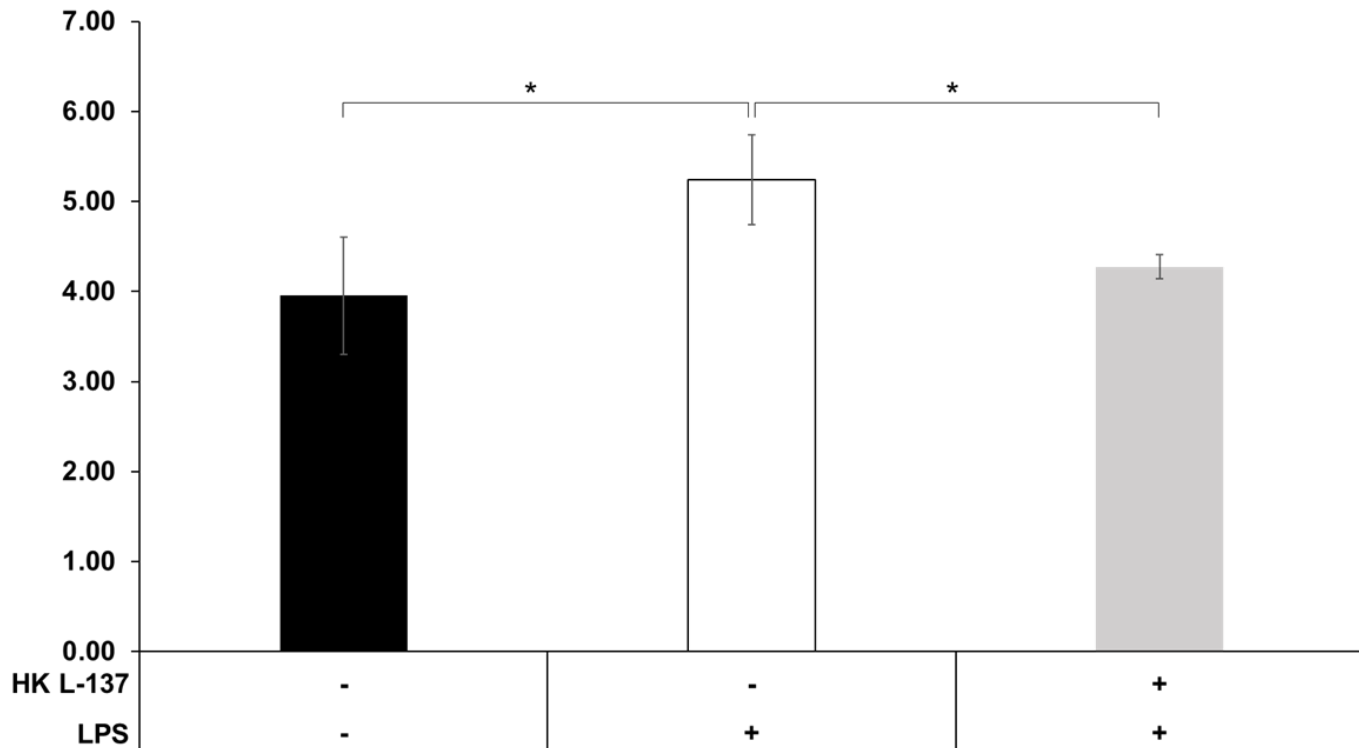

Supplement: Supplementary file 4 — Supplementary Figure S3. [file 41598_2024_62657_MOESM4_ESM.pdf]
