# Supplementary material for: Beneficial effect of heat-killed Lactiplantibacillus plantarum L-137 on intestinal barrier function of rat small intestinal epithelial cells
Source: Sci Rep. 2024 May 29;14:12319. doi: 10.1038/s41598-024-62657-0 (PMC11136994; doi:10.1038/s41598-024-62657-0)
Supplement: Supplementary file 5 — Supplementary Legends. [file 41598_2024_62657_MOESM5_ESM.docx]

**Additional material**

Additional file 1: Supplemental table Real time PCR primers for analysis.doc

Supplemental table Real time PCR primers for analysis.

Additional file 2: Supplemental figure S1 Full-length images of representative bands shown in Figure 3.pdf

Supplemental figure S1 Full-length images of representative bands shown in Figure 3.

(A) ZO-1, (B) Occludin, (C) Total protein expression in rat IEC-6 cells treated with HK L-137 (500 μg/ml) for 24 hours was measured by Western blot analysis. Independent treatment was carried out in triplicate and images of representative bands are shown. The bands surrounded by a black line were trimmed and shown in the main text.

HK L-137, heat-killed *Lactiplantibacillus plantarum* L-137; ZO-1, zonula occludens-1

Additional file 3: Supplemental figure S2 Full-length images of representative bands shown in Figure 6.pdf

Supplemental figure S2 Full-length images of representative bands shown in Figure 6. (A) p-ERK/ERK, (B) p-AMPK/AMPK, (C) p-Akt/Akt levels were measured in rat IEC-6 cells treated with or without the inhibitors of ERK1/2 (PD98059, 25 μM), AMPK (dorsomorphin, 20 μM), or Akt (LY294002, 5 μM) for 1 hour, followed by 15 minutes of treatment with HK L-137 (500 μg/ml). Independent treatment was carried out in triplicate and images of representative bands are shown. The bands surrounded by a black line were trimmed and shown in the main text, but the positions of the bands in the L-137 alone and inhibitor alone treatments are shown interchangeably.

AMPK, AMP-activated protein kinase; DO, dorsomorphin; ERK, extracellular signal-regulated kinase; HK L-137, heat-killed *Lactiplantibacillus plantarum* L-137; LY, LY294002; PD, PD98059; p-AMPK, phosphorylated AMP-activated protein kinase; p-ERK, phosphorylated extracellular signal-regulated kinase

Additional file 4: Supplemental figure S3 Effect of HK L-137 on LPS-induced increase in FD-4 permeability of IEC-6 cells.

FD-4 was added to rat IEC-6 cells treated with HK L-137 (500 μg/ml) for 24 hours and then with LPS (Escherichia coli O127:B8, Sigma-Aldrich, L3129, 100 μg/ml) for 26 hours, and fluorescence intensity was measured 5 hours later. Excitation wavelength 490 nm, emission wavelength 520 nm. The figure shows the beneficial effect of HK L-137 on intestinal barrier dysfunction. Means ± S.D., *n* = 4, Newman-Keuls test, **P* < 0.05

FD-4, fluorescein isothiocyanate-dextran; HK L-137, heat-killed *Lactiplantibacillus plantarum* L-137; LPS, lipopolysaccharide
